# Clinical Trials of a Stroke Rehabilitation Trainer Employing a Speed-Adapted Treadmill

**DOI:** 10.3390/s25185834

**Published:** 2025-09-18

**Authors:** Fu-Cheng Wang, Szu-Fu Chen, Pen-Ning Yu, Yin Keat Tan, Hsin Ti Cheng, Chia-Wei Chang, Lin-Yen Cheng, Yen-Chang Chien, Lik-Kang Koo

**Affiliations:** 1 Department of Mechanical Engineering, National Taiwan University, Taipei 106, Taiwan; r10522851@ntu.edu.tw (Y.K.T.); r11522858@ntu.edu.tw (H.T.C.); r12522849@ntu.edu.tw (C.-W.C.); 2 Department of Physical Medicine and Rehabilitation, Cheng Hsin General Hospital, Taipei 112, Taiwan; a09830886@gmail.com (L.-Y.C.); cute29554416@gmail.com (Y.-C.C.); josephlkkoo@gmail.com (L.-K.K.); 3 Department of Physiology and Biophysics, National Defense Medical Center, Taipei 114, Taiwan; 4 Department of Mechanical Engineering, National Cheng-Kung University, Tainan 701, Taiwan; penningyu@gs.ncku.edu.tw

**Keywords:** rehabilitation, stroke, gait, trainer, treadmill, control, robust, neuro-developmental treatment

## Abstract

We propose a speed-adapted treadmill that can be incorporated into a rehabilitation trainer that applies neurodevelopmental treatment (NDT) for patients with stroke. NDT practice is effective for post-stroke patients, but its requirement for therapists’ participation can limit the patients’ rehabilitation during the golden period of recovery. Previous studies have proposed a trainer that can automatically reiterate therapists’ interventions. However, that trainer employed a constant-speed treadmill, which required the users to frequently adjust their walking speeds during rehabilitation. This paper develops a speed-adapted treadmill that can regulate the treadmill motor to maintain the subject’s position during the training process. First, we derive models of the treadmill and cable motors through experiments. Then, we design robust controls for the two systems and simplify them as proportional-integral-derivative controllers for hardware implementation. Finally, we integrate the system and invite healthy and stroke subjects to participate in clinical experiments. Among ten stroke subjects, all subjects’ walking speeds and nine subjects’ stride lengths were improved, while eight subjects showed improvement in the swing-phase asymmetry and pelvic rotation after receiving the NDT rehabilitation employing the speed-adapted treadmill. Our findings indicate that the NDT trainer effectively enhances users’ gait characteristics, including swing-phase symmetry, pelvic rotation, walking speed, and stride length.

## 1. Introduction

The rapid pace of modern society has led to a fast-paced, stressful, and highly efficient lifestyle. This shift has caused people to gradually overlook the importance of physical health and develop numerous unhealthy habits. These habits have contributed to stroke becoming a common disease in the world and, together with other cerebrovascular diseases, now represent the second leading cause of death [1]. Stroke often leads to multiple complications and long-term impairments, resulting in significant functional disability. Indeed, a cohort study reports that up to 75% of stroke survivors experience residual impairment and disability, and 15–30% report significant, potentially permanent limitations [2]. These consequences substantially reduce patients’ ability to perform daily activities, particularly walking. For this reason, many rehabilitation devices now focus on restoring the walking capabilities of post-stroke patients. For instance, a Cochrane review concluded that physiotherapy supplemented with electromechanical or robotic gait training devices will likely improve walking independence compared with no intervention, especially when initiated early after stroke [3].

Colombo et al. [4] designed a robotic exoskeleton for patients with foot paralysis, and Yano et al. [5] developed a trainer to enhance stability on uneven surfaces. Roy et al. [6] further introduced a lightweight ankle robot. Although these devices provide structured guidance through predefined movements, they primarily serve as walking aids rather than platforms for autonomous gait training. Their assistance involved passive movement of the extremities without active patient engagement, thereby limiting opportunities for motor learning [7].

Neuro-developmental treatment (NDT), also named Bobath therapy, emphasizes active participation by focusing on the patient’s balance and trunk control during dynamic movement assisted by the therapist [8]. One study demonstrated that a Bobath-based trunk training program significantly improved trunk balance and control, functional capacity, and gaits in patients with stroke, with outcomes surpassing those of conventional exercise-based interventions in various clinical measures [9]. These findings underscore NDT’s relevance in promoting active motor recovery beyond the confines of passive robotic assistance.

NDT cues stroke patients to start gait cycles at critical points, thereby enabling them to engage in muscle activation and weight redistribution [7,8,10]. Pathak et al. [11] discovered that chronic stroke patients treated using NDT rehabilitation principles showed significantly enhanced balance, mobility, and gait performance. Through consistent NDT training sessions, patients could progressively regain their walking proficiency by motor learning [12]. However, although conventional NDT rehabilitation can improve functional mobility and gait performance in stroke patients, it requires substantial expenditures of effort and time by therapists. Consequently, a lack of therapists’ assistance might result in insufficient rehabilitation treatments during the patients’ golden period for recovery. For this reason, Wang et al. [13,14] proposed NDT trainers, which can automatically repeat an NDT intervention, thereby alleviating therapists’ workloads and extending patients’ training time. They studied the therapists’ intervention patterns in manual NDT and concluded that the therapists cued the subjects’ anterior superior iliac spines (ASISs) when perceiving opposite heel strikes (HSs). Hence, real-time recognition of the HS events was deemed essential for NDT rehabilitation.

The recognition of gait patterns has since attracted intensive research attention. For example, Yun et al. [15] integrated acceleration data to correct for drift in precise position estimation. Rebula et al. [16] proposed algorithms to estimate foot placement and variability from inertial data. Aminian et al. [17] developed a system to identify spatiotemporal parameters in walking. Wang et al. [13,14] utilized visual recognition to catch HS events to develop a stationary NDT trainer. The essential gait events can also be recognized using gait data obtained from inertial measurement units (IMUs) [18,19]. A subsequently developed Long Short-Term Memory (LSTM) model was capable of detecting HSs with an accuracy exceeding 99% [20]. Experiments employing gait recognition techniques confirmed the NDT trainer’s ability to improve users’ longitudinal gait performance [13,14]. The NDT intervention patterns were then modified to improve pelvic rotation and lateral movements [18,21,22,23].

This paper applies these modified NDT intervention methods to a stationary NDT trainer. When using a stationary NDT trainer [13,14], patients frequently walk on a constant-speed treadmill and need to adjust their walking speeds. This might significantly affect the subjects’ motions, especially among stroke patients, who usually have inconsistent walking speeds due to paralysis in the affected leg [22,24]. Typically, the stride length of the affected leg is roughly half that of the unaffected leg. At the beginning of the experiments, patients need to establish their most comfortable speed by walking on the treadmill. These extra steps prolong the training periods and might exhaust patients physically and mentally. To address this issue, this paper proposes a speed-control treadmill that enables patients to walk at their preferred speeds during the treatments.

Speed-adapted treadmills have previously been applied for rehabilitation. For example, Gembalczyk et al. [25] proposed a speed-control treadmill that adjusted belt speeds using feedback control. Von et al. [26] measured the horizontal interaction forces at the user’s trunk to control the treadmill belt speed. Auralius et al. [27] utilized a sonar sensor that fed back the subject’s position to control the treadmill motor. In this paper, we applied an infrared range (IR) sensor to provide feedback regarding the user’s location, and we used a robust proportional-integral-derivative (PID) compensator to regulate the treadmill speed to maintain the user’s position during the NDT training. We also recruited subjects to conduct clinical experiments with a stationary NDT trainer employing the speed-controlled treadmill. Our results indicated that the speed-controlled treadmill effectively improved the subjects’ gait performance, including longitudinal symmetry, pelvic rotation, walking speed, and stride length, during NDT training.

## 2. Materials and Methods

**Figure 1:**
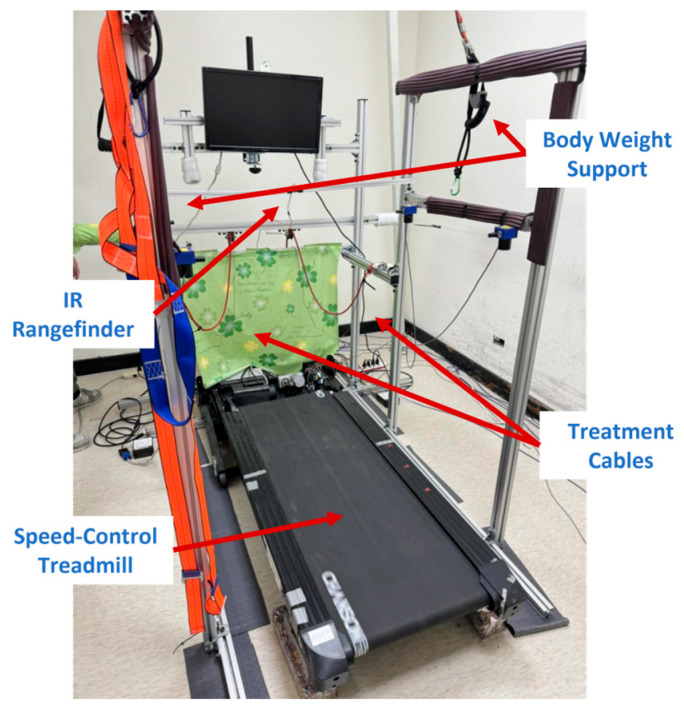
The stationary NDT trainer.

The stationary NDT trainer, shown in Figure 1, comprises motion capture, expert, cable control, and treadmill control systems. The motion capture system uses two IMUs [28] to detect HSs, which activate the expert and cable control systems. One IMU on the user’s waist is also equipped to measure pelvic rotation. The expert system repeats the intervention patterns based on the IMU data, while the cable control system realizes the NDT interventions. The treadmill control system is equipped with an IR sensor, which provides feedback regarding the user’s position to regulate the treadmill motor, thereby maintaining the user at a preset position on the treadmill during the NDT rehabilitation. The system specifications are illustrated in Appendix A [25,26,27,28,29,30,31,32].

**Figure 2:**
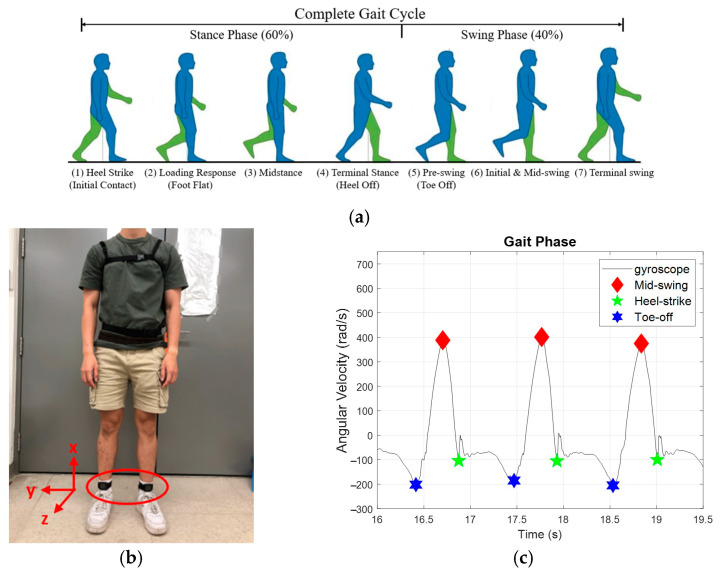
Gait patterns. (**a**) the gait cycles. (**b**) IMUs attachments. (**c**) gait response.

As shown in Figure 2a, human gaits are periodic, consisting of three important events—toe-off, mid-swing, and HS [29]. We applied two IMUs, as illustrated in Figure 2b, to measure the subjects’ kinematic data. For example, the standard angular velocity during walking is shown in Figure 2c, where the highest angular velocity in the gait cycle occurs at mid-swing. In contrast, the first trough after mid-swing represents the HS.

We analyzed clinical NDT sessions [13,23] and concluded that the therapists cued the patients’ opposite ASIS when observing the HS events. Therefore, we developed an LSTM model that would detect HSs [20] to trigger the expert and cable control systems for rehabilitation intervention, where three groups of subjects tested the LSTM model: the elderly, patients with stroke, and patients with Parkinson’s disease. The results indicated accuracy exceeding 98% with a maximum error of less than one sample (0.04 s) [20].

### 2.1. NDT Intervention

**Figure 3:**
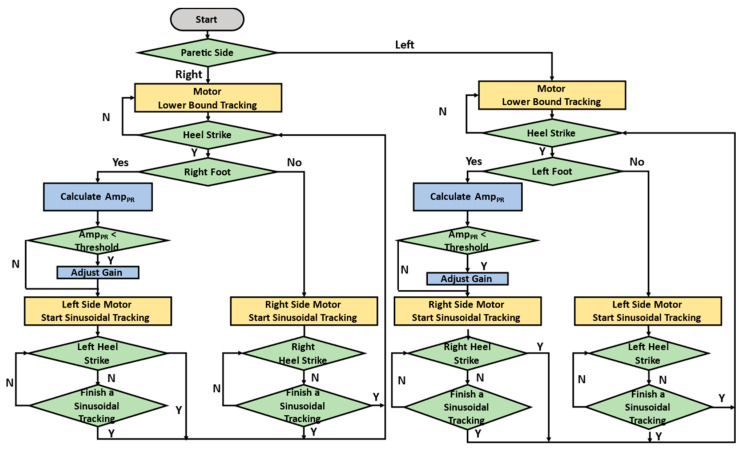
NDT intervention processes.

We applied the NDT intervention [18,23], where the cable motor controls a force command on the opposite side

(1) $$F(t)=\frac{\left(MF_{\max}-F_{\min}\right)}{2}\times \sin(2\pi f)+\frac{\left(MF_{\max}+F_{\min}\right)}{2},$$

when detecting HS [18]. *M* is the force-amplified factor that increases the intervention force on the non-paretic side [13,14]. $F_{\max}$ and $F_{\min}$ represent the maximal and minimal forces, respectively. *f* is the cuing frequency. We set $F_{\max}$ = 6 lbs. (26.67 N), $F_{\min}$ = 1 lb. (4.44 N), *f* = 1 Hz, and *M* = 1.5 when the pelvic rotation is less than $12^{\circ }$ [18,23]. The overall intervention processes are illustrated in Figure 3. The detection system recognizes HS events and pelvic rotation, while the expert system, upon detecting an HS, modifies the force command to regulate the cable control system.

For example, consider a patient with right hemiplegia. The system captured the gait cycle using IMUs attached to the subject’s legs. Before the right HS, the left motor maintained a fixed tension of 4.44 N to keep the cable straight. Upon detecting the right HS, the left motor began to track the command of (1), where *M* = 1.5 when the pelvic rotation is less than $12^{\circ }$. Before the left HS, the right motor maintained a fixed tension of 4.44 N to keep the cable straight and began to track the command of (1) with *M* = 1 when detecting the left HS.

### 2.2. Performance Evaluation

We evaluated the effects of rehabilitation using the subjects’ longitudinal gait symmetry, pelvic rotation, walking velocity, and stride length.

**Figure 4:**
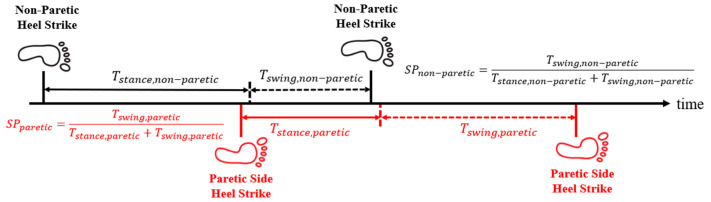
Illustration of the swing phases.

**Figure 5:**
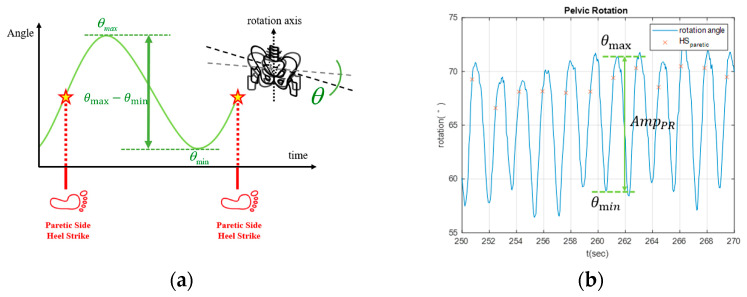
Pelvic rotation. (**a**) schematic diagram. (**b**) Pelvic rotation of a stroke patient with right hemiplegia.

**Figure 6:**
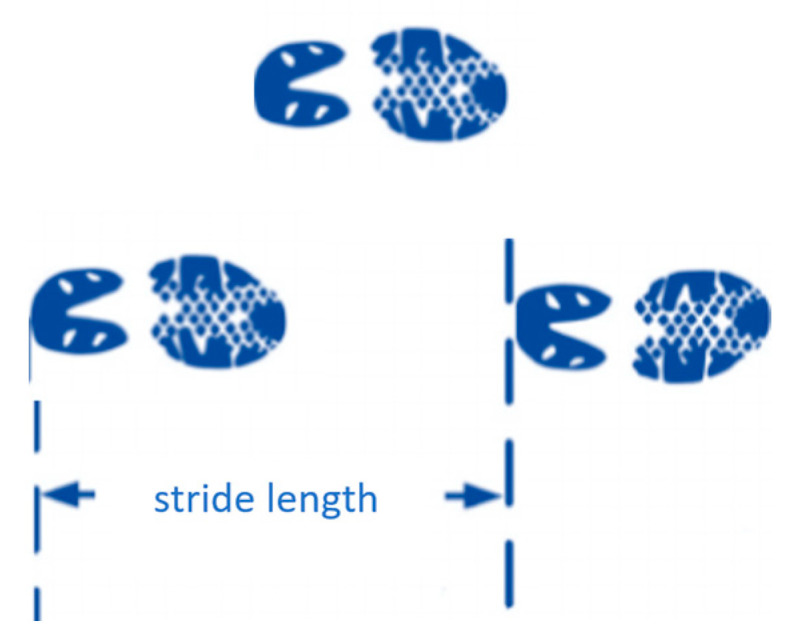
Schematic diagram of stride length calculation.

(1) Longitudinal symmetry: Longitudinal symmetry is a performance index for subjects’ forward walking. We defined the ratio of swinging time as follows:
  (2) $$SP=\frac{T_{SW}}{T_{Gait}}\times 100\%$$
  where $T_{SW}$ and $T_{Gait}$ represent the time durations of the swinging phase (from toe-off to HS) and the gait cycle, respectively. For healthy individuals, $T_{SW}$ is typically 40% of $T_{Gait}$ with bilateral symmetry. However, stroke patients often have weak muscle endurance on the affected side and develop asymmetric gait patterns, where the sound side usually has a shorter swing phase due to hemiparesis [30,31], as shown in Figure 4. Hence, we define:
  (3) $$Asym_{SP}=\frac{SP_{paretic}-SP_{non-paretic}}{SP_{paretic}}\times 100\%$$
  where $SP_{paretic}$ and $SP_{non-paretic}$ denote the swing phases of the paretic and non-paretic limbs, respectively.
(2) Pelvic rotation: Effective pelvic rotation contributes to patients’ walking stability and reduces the risk of falls. Hence, we define the amplitude of pelvic rotation as follows:
  (4) $$Amp_{PR}=\theta_{\max}-\theta_{\min}$$
  where $\theta_{\max}$ and $\theta_{\min}$ correspond to the maximum and minimum rotational angles between two consecutive HSs, respectively (see Figure 5a). During walking, the stroke patients’ muscle strength on the affected side is usually insufficient to provide stable support for stepping. Consequently, their pelvic rotation noticeably decreases. For example, the pelvic rotation of a patient with right hemiplegia is illustrated in Figure 5b, where $Amp_{PR}$ at some gait cycles was less than a threshold of $12^{\circ }$, resulting in increases in the cuing forces [23].
(3) Walking speed: Walking speed is widely recognized as a primary measure of gait and is considered a key clinical indicator of functional ability in individuals following stroke. It reflects the integrated function of the neuromuscular, cardiovascular, and balance systems. Comfortable walking speed is highly sensitive to post-stroke deficits, and affected individuals consistently demonstrate slower speeds than healthy adults [32]. This slow gait has been linked to restrictions in community ambulation, loss of independence, a higher risk of falls, and a lower quality of life [33]. The 6-Minute and 10-Meter walk tests, as standardized measures in clinical practice, are frequently used to assess gait speed [33]. Improvements in gait speed have been associated with greater balance, less variability in gait, and better dynamic stability [32]. In rehabilitation, gait speed is an important endpoint and therapeutic goal. Strategies such as treadmill walking exercise, robot-assisted gait, and speed-related locomotor training have enhanced the gait speed and improved residual motor function [32]. Gait speed also has a predictive value concerning length of stay estimates, discharge destination, or subsequent ambulatory status [33,34]. Therefore, walking speed assessment and training should be considered central components of stroke rehabilitation.
(3) Stride length: The distance between consecutive HSs of the ipsilateral limbs (see Figure 6) is a key spatiotemporal parameter in gait analysis. Gait patterns following stroke frequently demonstrate stride length asymmetry, wherein the paretic limb generated shorter strides than the non-paretic limb. This asymmetry reflects impairments in limb propulsion, swing, balance control, and coordination and is linked to increased energy expenditure, reduced gait efficiency, and a higher fall risk [35,36]. Greater asymmetry also predicts extended rehabilitation stays and delayed recovery [36]. Importantly, asymmetry may represent an adaptive strategy. For example, reduced hip flexor activity on the paretic limb may lead to overreliance on the non-paretic limb, resulting in biomechanical inefficiency and abnormal loading patterns [35]. Commonly applied rehabilitation interventions include step-length training with fast walking training [37,38], split-belt treadmill walking [39,40], and functional electrical stimulation of the plantarflexors [38]. Stride length is a key determinant of walking speed [41]. Among stroke patients, a limited capacity to increase stride length may hinder improvements in gait speed and instead result in compensatory increases in cadence [41]. This limitation affects energy efficiency and impedes progress toward a more stable and symmetrical gait [42]. Consequently, stride length should be evaluated as an isolated measure of asymmetry and as a contributor to global locomotor capacity. Integrating step-length measurements into rehabilitation protocols may enable targeted therapy for functional ambulation.

### 2.3. Treadmill Speed Control

In previous NDT experiments [13], we observed that the subjects needed to make frequent adjustments to their walking paces according to the treadmill velocities. Stroke patients’ walking speeds are highly uneven and varied because of hemiparesis; hence, the constant-speed treadmill might jeopardize their movements and make their walks unnatural, while also causing physical and mental exhaustion during NDT training. This paper proposes a speed-control treadmill that adjusts the motor based on the subject’s walking speed, thereby allowing the subject to maintain a set position when undergoing NDT rehabilitation.

**Figure 7:**
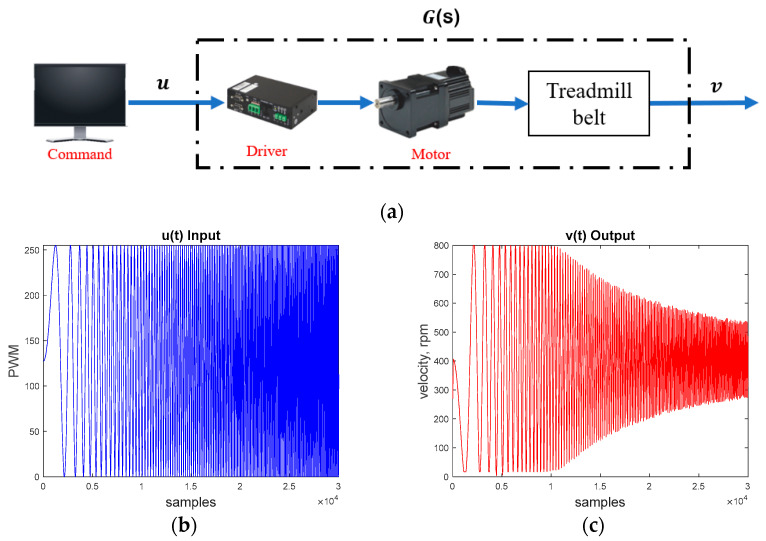
Treadmill motor system. (**a**) System identification diagram. (**b**) PWM command signal *u*. (**c**) Output velocity signal *v*.

We first derived the system’s transfer function *G*(*s*) by experiments, as shown in Figure 7a. We generated a swept sinusoidal pulse width modulation (PWM) signal (*u*) (see Figure 7b) to drive the treadmill motor. The PWM signal ranged from 0 to 255, where 255 corresponded to the motor’s maximum speed of 800 rpm (equivalent to a maximum treadmill velocity of 0.92 m/s). We then measured the system output velocity (*v*) from the motor encoder (see Figure 7c). Finally, we applied the MATLAB 2020a command *tfest* to acquire the system models. Considering the system uncertainties and disturbances, we conducted the experiments 10 times to derive the following 10 models:

(5) $$\begin{array}{c} G_{1}(s)=\frac{51.98}{s^{2}+6.86s+19.35},\;G_{2}(s)=\frac{52.58}{s^{2}+6.89+19.45},\\ G_{3}(s)=\frac{52.22}{s^{2}+6.89s+19.43},\;G_{4}(s)=\frac{52.25}{s^{2}+6.89s+19.44},\\ G_{5}(s)=\frac{52.27}{s^{2}+6.89s+19.44},\;G_{6}(s)=\frac{52.14}{s^{2}+6.87s+19.40},\\ G_{7}(s)=\frac{52.31}{s^{2}+6.90s+19.46},\;G_{8}(s)=\frac{51.21}{s^{2}+6.89s+19.43},\\ G_{9}(s)=\frac{52.13}{s^{2}+6.87s+19.40},\;G_{10}(s)=\frac{52.21}{s^{2}+6.88s+19.43}. \end{array}$$

We then selected a nominal plant $G_{0}=G_{6}$ from (5), based on their gaps [43], where $G_{6}$ gave a minimal gap of $\delta (G_{6},G_{i})\leq 0.0015,\;\forall i=1,\;2,\;\ldots ,\;10$.

**Figure 8:**
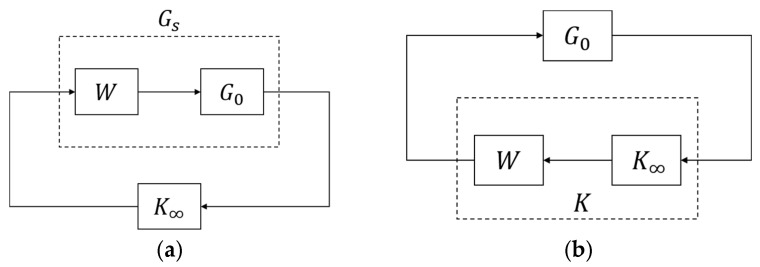
Control design. (**a**) $H_{\infty }$ loop-shaping control design. (**b**) Control implementation.

We applied loop-shaping techniques [44] (see Figure 8a), with the weighting:

(6) $$W(s)=\frac{\left(3s+5)(s+1\right)}{s},$$

to design a standard robust controller:

(7) $$K_{\infty }(s)=\frac{7.42\times 10^{4}s^{2}+1.92\times 10^{5}s+1.31\times 10^{5}}{s^{3}+7.64\times 10^{4}s^{2}+2.04\times 10^{5}s+1.27\times 10^{5}},$$

by the Matlab command *ncfsyn*. The system remains internally stable if and only if $b(G_{0},K)\geq \delta$, where δ is the system gap and $b(G_{0},K)$ is the stability margin [45]. For example, the controller in (7) has a stability margin $b(G_{0}W,K_{\infty })=0.7163$ > 0.0015, so system stability is guaranteed. We then implement $K(s)=W(s)K_{\infty }(s)$ to control *G*_0_(*s*) (see Figure 8b).

Because the standard robust control K(s) was fourth order, we applied particle swarm optimization (PSO) [46] algorithms to simplify it as the following robust PID controller:

(8) $$C(s)=K_{P}+\frac{K_{I}}{s}+K_{D}s$$

for its simplicity and preference in industrial applications.

PSO is inspired by the concept of biological swarms and designed to emulate stochastic optimization, where each particle represents a candidate in the search. Initially, particles are randomly distributed throughout the problem domain and iteratively update their velocities and positions to converge toward the optimal solutions. In each iteration, the new velocity and position are computed as follows:

(9) $$V_{i}^{k+1}=\;W\cdot V_{i}^{k}+\;C_{1}\cdot rand_{1}\cdot \left(Pbest_{i}-\;X_{i}\right)+\;C_{2}\cdot rand_{2}\cdot \left(Gbest-X_{i}\right),$$

(10) $$X_{i}^{k+1}=X_{i}^{k}+V_{i}^{k+1},$$

where *X_i_^k^* and *X_i_^k+^*^1^ stand for the present and new positions, while *V_i_^k^* and *V_i_^k+^*^1^ symbolize the present and new velocities. $Pbest_{i}$ represents the best position found by each individual particle, and Gbest represents the best global position discovered by the swarm. The particles are updated through the weight *W*, the learning coefficients $C_{1}\;\mathrm{and}\;C_{2}$, and the random numbers $rand_{1}\in \left[0,1\right]\;\mathrm{and}\;rand_{2}\in \left[0,1\right]$. We set $C_{1}\;=0.2\;\mathrm{and}\;C_{2}=0.2$ and let *W* decreases linearly from 0.6 to 0.4 over the iterations:

(11) $$W=(0.6-0.4)\times \frac{iter-i}{iter}+0.4$$

where *iter* = 50 is the number of iterations, while *i* represents the present iteration index.

We applied PSO to design a robust PID controller using the following fitness function:

(12) $$F(K_{P},K_{I},K_{D})=\sum_{i=1}^{5}\omega_{i}\cdot \bar{J_{i}}$$

where $\omega_{i}$ is the weighting for performance index $\bar{J_{i}}$, representing the similarity of the robust control K(s) and the robust PID control C(s). $\bar{J_{i}}$ is defined as:

(13) $$\bar{J_{i}}={\left(\frac{J_{i}^{PID}}{J_{i}^{Robust}}-1\right)}^{2}$$

where $J_{i}^{PID}$ and $J_{i}^{Robust}$ represent the performance index $J_{i}$ of the system employing the robust PID and standard robust controllers, respectively.

Consider the following five performance indexes:

1. Stability margin $J_{1}$.
2. Root mean square error (RMSE) $J_{2}$ of step-responses.
3. Settling time $J_{3}$ of step-responses.
4. Overshoot $J_{4}$ of step responses.
5. Rise time $J_{5}$ of step response.

**Figure 9:**
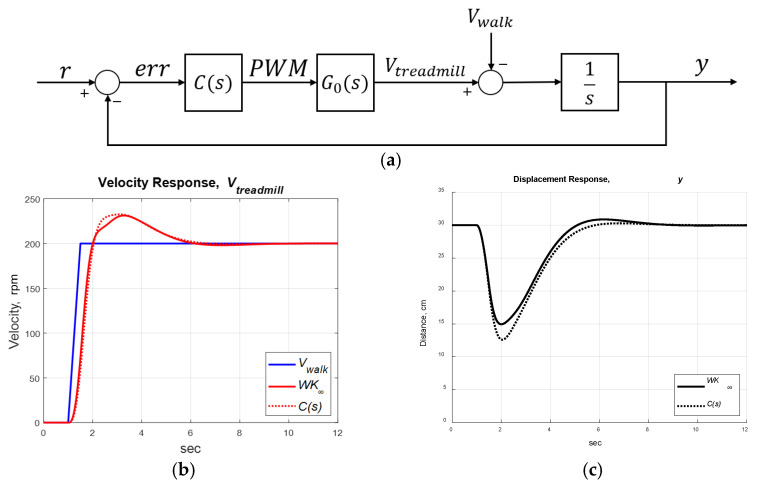

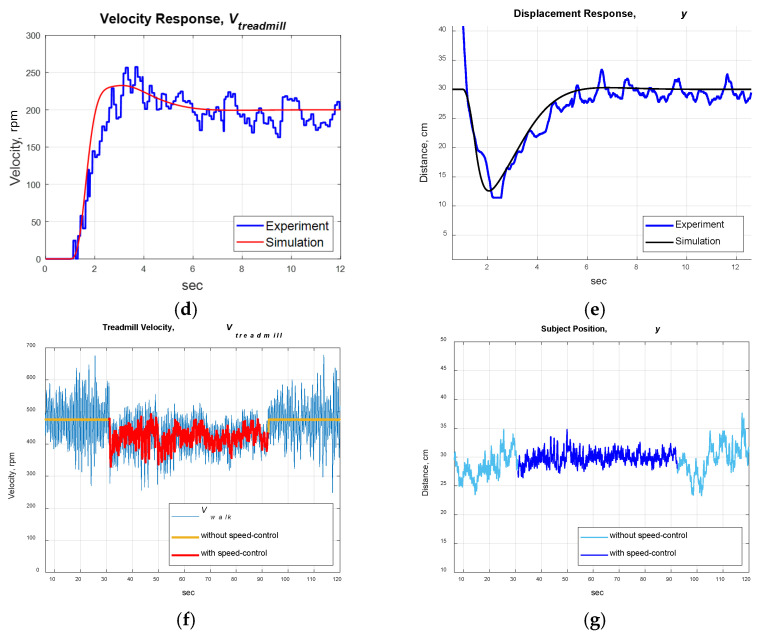
Treadmill control system. (**a**) System block diagram. (**b**) Treadmill velocity responses (simulation). (**c**) Subject’s position responses (simulation). (**d**) Treadmill velocity responses (experiment). (**e**) Subject’s position responses (experiment). (**f**) Treadmill velocity responses (experiment with control off/on/off). (**g**) Subject’s position responses (experiment with control off/on/off).

Setting $\omega_{i}=1$ for i=1, 2, …, 5, the robust PID controller was derived as in Equation (8) with $K_{P}=7.191$, $K_{I}=4.242$ and $K_{D}=2.036$, which gave a stability margin of $b(G_{0},C)=0.3033$. We implemented the standard robust and robust PID controllers (see Figure 9a) and set *r* = 30 cm, which meant that the subject should be 30 cm in front of the IR rangefinder, around the middle of the treadmill. The subject’s walking speed ($V_{walk}$) was treated as a disturbance. The control objective was to maintain the user’s position at a fixed distance of 30 cm from the IR range finder, regardless of external disturbances. The simulations are compared in Figure 9b,c, where C(s) provided similar responses as the robust control K(s). Therefore, we implemented the robust PID control C(s) for experiments.

We implemented the designed robust PID control on the treadmill and invited a healthy subject to participate in the experiments. The subject initially stood on the treadmill with zero velocity. When the subject began to walk on the treadmill, the IR sensor provided feedback to maintain the position y→r=30. The experimental responses were similar to those of the simulation (see Figure 9d,e). We can transfer the motor’s rotational speed ω (rpm) to the belt’s speed *V* (m/s) by

(14) V=ωR⋅πD/60

where *R* = 0.5 is the gear ratio of the motor and *D* = 0.044 m is the diameter of the belt pulley. The distance between the subject and the IR sensor oscillated around 30 cm due to the disturbances $V_{walk}$, maintaining the subject at y≈30 cm.

The advantages of the speed-adapted treadmill are demonstrated in Figure 9f,g, where the speed control was off at t∈[0, 30] s, then turned on at t∈[30, 90] s, and off again at t>90 s. Without speed control, the treadmill maintained a constant speed, so the users needed to adjust their walking speeds to avoid exceeding the treadmill’s ends. In contrast, the speed-control treadmill adjusted its velocity to maintain the user’s position at about 30 cm from the IR sensor. The RMSE of the subject’s position was 2.68 cm at t∈[0, 30] s, then improved to 1.13 cm at t∈[30, 90] s, and worsened to 2.71 cm at t>90 s.

Stroke patients often experience uneven and unstable walking speeds because of insufficient muscle strength on the affected side. Therefore, a speed-controlled treadmill can adapt to the walking pace of stroke patients, enabling them to walk comfortably on the treadmill. That is, stroke patients can focus on undergoing NDT training at their most comfortable speeds with the assistance of a speed-controlled treadmill.

### 2.4. Cable Force Control

**Figure 10:**
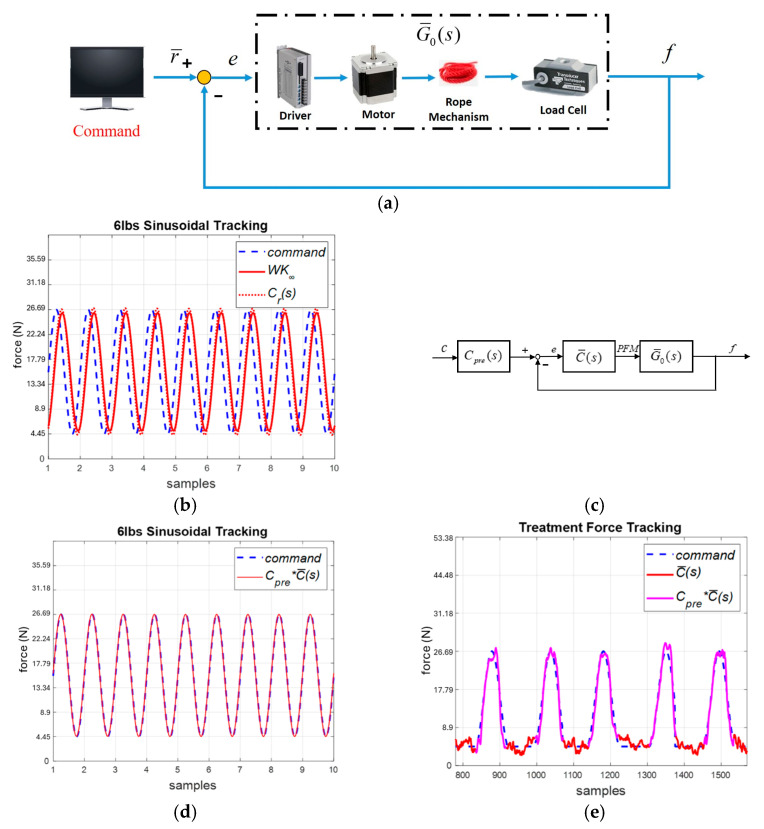
Cable motor system. (**a**) System identification. (**b**) System response by the standard robust and robust PID controllers (simulation). (**c**) System with a pre-compensator. (**d**) Sinusoidal force tracking with pre-compensator (simulation). (**e**) Experimental responses.

The cable force control system is shown in Figure 10a. We generated a swept sinusoidal force signal $\bar{r}$ and measured the output force *f* by load cells to derive the system model ${\bar{G}}_{0}(s)$. Similarly, we conducted the identification experiments 10 times and selected the following nominal plant:

(15) $${\bar{G}}_{0}(s)=\frac{-47.32s+484.5}{s^{2}+55.87s+118.1},$$

which gave a minimal system gap of δ=0.059.

We applied the weighted plant ${\bar{G}}_{s}(s)={\bar{G}}_{0}(s)\bar{W}(s)$ with:

$$\bar{W}(s)=\frac{2s+4}{s(0.02s+1)},$$

to design the following loop-shaping controller:

(16) $${\bar{K}}_{\infty }(s)=\frac{1.37\times 10^{4}s^{3}+1.39\times 10^{6}s^{2}+3.35\times 10^{7}s-6.35\times 10^{7}}{s^{4}+3939s^{3}+6.68\times 10^{5}s^{2}+1.17\times 10^{8}s+2.31\times 10^{8}},$$

by the Matlab command *ncfsyn.* The stability margin $b({\bar{G}}_{0}\bar{W},{\bar{K}}_{\infty })=0.2651$ > 0.059 so that system stability is guaranteed.

The sixth-order weighted controller $\bar{K}(s)=\bar{W}(s){\bar{K}}_{\infty }(s)$ was simplified into the following robust PID controller for practical implementation:

(17) $$\bar{C}(s)={\bar{K}}_{P}+\frac{{\bar{K}}_{I}}{s}+{\bar{K}}_{D}s,$$

where ${\bar{K}}_{P}=0.387$, ${\bar{K}}_{I}=1.053$ and ${\bar{K}}_{D}=0.255$. We implemented $\bar{K}$ and $\bar{C}$. The system responses are compared in Figure 10b, where the robust PID control $\bar{C}$ provided similar responses to those obtained with $\bar{K}$. However, this was associated with a phase lag of about $37^{\circ }$. Hence, we applied the following pre-compensator, as shown in Figure 10c, to eliminate the phase lag:

(18) $$C_{pre}(s)=\frac{0.1512s+0.315}{0.03s+1}.$$

As shown in Figure 10d, the system responses can track the input commands. Finally, we conducted experiments to verify the cable control system. Given a force command, the system could successfully track the intervention patterns, as shown in Figure 10e, with an RMSE of than 0.5 lb. Therefore, we applied the trainer for NDT rehabilitation training.

Before the clinical experiments, we invited four healthy subjects to test the trainer with a constant-speed treadmill and a speed-adapted treadmill. The results are shown in Appendix B, where all subjects’ walking speed and stride length were improved, while three subjects showed improvements in longitudinal symmetry and pelvic rotation. The NDT training with the speed-adapted treadmill is more effective than a constant-speed treadmill.

### 2.5. NDT Rehabilitation Experiments

We integrated the NDT trainer and invited healthy and stroke subjects to participate in experiments. The demographic data are presented in Appendix C. The Institutional Review Board approved the experiments at Cheng Hsin General Hospital. All subjects signed written informed consent before the experiments.

Each subject participated in the experiments at three stages: A, B, and $\bar{A}$, where A indicates the pre-treatment phase, B represents the treatment phase, and $\bar{A}$ corresponds to the post-treatment phase. All subjects walked on the speed-controlled treadmill without intervention for approximately 100 s at stage A, followed by about 200 s with NDT intervention at stage B, and finally, with no intervention at stage $\bar{A}$ for approximately 100 s. No adverse events occurred in either the stroke or healthy cohorts during or after the experiments.

## 3. Results

Five female and five male healthy subjects, aged 22 to 25, participated in the experiments. The demographic data are presented in Appendix C. They wore a rehabilitation gaiter to limit knee flexion, mimicking the typical extensor synergy patterns after a stroke. Their gait data and performance indexes are shown in Appendix D. The improvement ratios are illustrated in Table 1, regarding the asymmetry of swing phases, pelvic rotation, walking speed, and stride length. We estimated the walking speed by the motor rotational speed according to (14), because the IR signal variation was much smaller than the walking distance during the stages and can be neglected. In addition, we calculated the stride lengths by taking the integrals of the walking speed because the IR signal variation was much smaller than the walking distance during the stages.

Comparison of Stage B and Stage A showed that all subjects exhibited improvements in $Asym_{SP}$, while $Amp_{PR}$ was increased in eight subjects. These responses indicated that the subjects achieved a more stable pelvic movement when receiving the NDT rehabilitation. Finally, all subjects’ walking speeds and stride lengths were improved. Comparison of Stage $\bar{A}$ and Stage A revealed improvement in nine subjects in $Asym_{SP}$, and an increase in $Amp_{PR}$ in nine subjects, indicating that their pelvises were conserving energy more effectively during walking. Overall, all subjects showed improvements in walking speed and stride length. Based on these results, the NDT intervention with the speed-control treadmill was deemed safe and to have a positive impact on stroke rehabilitation.

We recruited four female and six male stroke participants, aged 41 to 64, with Brunnstrom Stage 3–4 hemiparesis and were able to independently stand and initiate treadmill walking without assistance. The demographic data are presented in Appendix C. All were in the chronic stage (>6 months post-onset). None used an assistive walking device, and individuals with unstable medical comorbidities were excluded, although stable concomitant stroke secondary prevention medications were permitted. For safety, all participants wore a harness during treadmill walking to prevent falls, without body-weight support.

The stroke subjects’ gait data and performance indices for all subjects are presented in Appendix D, while the proportions of individuals showing improvement are summarized in Table 2. Comparison of Stage B and Stage A revealed that nine subjects exhibited improvements in longitudinal symmetry, while eight subjects increased their pelvic rotation. Nine subjects’ walking speeds and eight subjects’ stride lengths were also improved. Comparison of Stage $\bar{A}$ and Stage A revealed that eight subjects showed improvement in the asymmetry of the swing phase, while eight subjects exhibited improvement in pelvic rotation. Overall, all subjects’ walking speeds and nine subjects’ stride lengths were improved. These results indicated that NDT training using a speed-controlled treadmill significantly improved gait performance in individuals with stroke.

## 4. Discussion

We proposed a speed-adapted treadmill for a stationary NDT trainer and conducted experiments to demonstrate its effects. First, we detected HS events in real time because the NDT principle involves cuing subjects’ ASIS when observing the opposite HS. Second, we developed a speed-control treadmill that can adjust the belt speed according to the user’s movement, thereby allowing the subjects to walk at their most comfortable paces while undergoing training. Third, we implemented the NDT intervention, which cued the opposite ASIS when detecting HS and increased the cuing forces on the sound side when observing insufficient pelvic rotation. Finally, subjects with stroke were recruited to participate in the experiments. Following the use of the gait trainer, most participants showed measurable enhancements in key gait parameters, including longitudinal symmetry, pelvic rotation, walking speed, and stride length.

Appendix D shows that the indices significantly varied among individuals due to different conditions. For example, the asymmetries of swing phase are 10.81~25.26%, pelvic rotations are 6.41~15.64°, the walking speeds are 249.54~575.04 rpm, and the stride lengths are 41.94~91.48 cm for healthy subjects at stage A. The corresponding indices for stroke patients at stage A are 11.26~46.17% 7.48~14.08°, 114.01~565.53 rpm, and 25.4~78.61 cm, respectively. Therefore, comparing within-subjects is challenging. Instead, we compared each subject’s indices at different stages.

While interpreting absolute magnitude changes is valuable, this feasibility study identified significant baseline heterogeneity within the stroke cohort, including variation in impairment severity, diverse gait patterns, and differing adaptation responses to treadmill and NDT cues. Additionally, the small sample size and the presence of outliers limit the reliability and interpretability of group mean changes or the application of a single magnitude for cross-sectional comparisons. Therefore, this study prioritized assessing within-subject changes across intervention stages and reporting the percentage of individuals exhibiting improvements in clinically meaningful gait parameters. This approach reduces the confounding effects of inter-subject variability and small sample size, offering more individualized insights into intervention responsiveness.

Regarding longitudinal asymmetry, we analyzed therapist actions and subject motions during conventional NDT training and concluded that therapists cued the subjects’ ASIS when observing HSs on the opposite side. First, the training stimulated leg movements and step cadences to improve the subjects’ longitudinal symmetry ($Asym_{SP}$) by activating leg motions. Second, the stroke patients’ deficiencies in hip, knee, and ankle flexions usually result in compensatory pelvic movements. In gait training, pelvic rotation is crucial for stride length extension and energy conservation. Hence, increasing the cuing forces serves to ameliorate pelvic rotation ($Amp_{PR}$). Third, stroke patients usually have muscle weakness and gait instability on the paralyzed side, resulting in a reduction in walking speed. NDT gait training can improve walking speeds ($V_{ave}$) and energy efficiency during ambulation in these patients by stimulating them to produce continuous gaits at key moments. Finally, stroke patients commonly exhibit impairment of the central nervous system’s regulation of muscle tone, resulting in sustained muscle tension and shortened stride lengths ($SL_{L}\;\mathrm{and}\;SL_{R}$) on both sides. NDT rehabilitation can enhance stride lengths by improving the control of muscular movements.

The speed-control treadmill offers substantial benefits by reducing the oscillation movement while also enhancing the efficiency of NDT training. Once subjects with stroke use the speed-control treadmill, they can walk at their most comfortable paces. Although stroke patients have uneven walking speeds, the speed-control treadmill can match the stroke patients’ walking pace by controlling the treadmill motor, thereby maintaining the subjects at a set location during the training. Based on the experimental results, significant improvements were achieved in longitudinal symmetry, pelvic rotation, walking speed, and stride length during and after the NDT intervention.

The 10 stroke patients participated in interviews after using the NDT trainer. They expressed appreciation for being able to walk at their preferred speeds during the automatic NDT rehabilitation. In addition, the speed-control treadmill gave them better mobility and posture when walking during the training.

Compared with previous studies, our work shares both similarities and key distinctions. For instance, a recent study using speed-dependent treadmill training with balance perturbation reported improvements in walking speed and stability among chronic stroke patients [5], and robotic gait training with body-weight support is more effective than conventional methods [6]. In contrast, our NDT-based, speed-adjusted treadmill does not rely on externally directed interventions but emphasizes active patient engagement and individualized adjustments. This approach is designed to foster motor learning rather than reduce the demand for active effort, which may account for the functional improvements observed in our patients.

In this study, all treadmill walking sessions were conducted in the rehabilitation laboratory and supervised by physiatrists. A safety harness, used without body weight unloading, was employed for all participants to prevent falls during gait training. Throughout all sessions, no adverse events were reported. Specifically, there were no falls or near falls, cardiovascular or neurological symptoms, or musculoskeletal injuries. Furthermore, there were no device-related issues, and no participants withdrew from the study prematurely. Most participants tolerated the sessions well, and the few who reported mild, temporary exercise-induced fatigue experienced spontaneous resolution without intervention.

Several limitations should be considered when interpreting the findings of this study. These include the small sample size, the single-center design, the absence of a randomized control group, and the short follow-up period, precluding a conclusion of long-term efficacy. Furthermore, participants were limited to Brunnstrom Stage 3–4, potentially restricting the generalizability of the results to individuals with milder or more severe disability. Acknowledging these limitations, we emphasize the need for larger, multicenter randomized cross-over studies with control conditions, multiple sessions, and long-term trials to confirm and extend our findings [47,48].

## 5. Conclusions

This paper describes the development of a speed-control treadmill and its integration with a stationary NDT trainer for stroke rehabilitation. First, we obtained the treadmill’s transfer functions through experiments and designed a robust PID controller to regulate the treadmill speed. The speed-control treadmill allows users to walk at their most comfortable pace during NDT training. It lets the subjects walk naturally and smooths the training procedure. Stroke patients, who usually develop irregular walking patterns, can now traverse the treadmill without needing to make continuous adjustments in their walking pace and can instead focus on the rehabilitation training to enhance their walking abilities. Second, we applied two wearable IMUs to the subject’s lower limbs to detect HS events, and one on the subject’s waist to measure pelvic rotations. The information received from these IMUs enabled modification of the NDT intervention to improve the subjects’ gait performance. Finally, we integrated the NDT intervention and the speed-controlled treadmill into an NDT trainer. The experimental results obtained using a group of healthy subjects confirmed the trainer’s safety and efficacy in enhancing gait performance. Subsequent clinical experiments with 10 recruited stroke patients confirmed the trainer’s effects in improving the patients’ walking capability in the four indices.

In the present study, our primary focus was on gait performance parameters, and we did not systematically collect physiological or perceptual measures. The benefits of reduced effort and fatigue can only be inferred from the observed gait improvements. Future studies should include quantitative measures like perceived exertion scales and cardiorespiratory responses to provide more substantial evidence for reduced effort and fatigue.

This study demonstrated the short-term rehabilitation effects of the NDT training employed the speed-adapted treadmill. Because stroke rehabilitation involves long-term processes, our future efforts will involve refining the trainer’s functionalities and conducting long-term clinical trials with larger stroke patient cohorts.

## Figures and Tables

**Table 1:** Subjects with improvements (healthy participants).

Performance Index	B Stage	$\bar{A}$ Stage
$Asym_{SP}$	10/10	9/10
$Amp_{PR}$	8/10	9/10
$V_{ave}$	10/10	10/10
$SL_{L},\;SL_{R}$	10/10, 10/10	10/10, 10/10

$V_{ave}$: average walking speed; $SL_{L}\;(SL_{R})$: average stride length on the left (right) side.

**Table 2:** Subjects with improvements (stroke patients).

Performance Index	B Stage	$\bar{A}$ Stage
$Asym_{SP}$	9/10	8/10
$Amp_{PR}$	8/10	8/10
$V_{ave}$	9/10	10/10
$SL_{L},\;SL_{R}$	8/10, 8/10	9/10, 9/10

$V_{ave}$: average walking speed; $SL_{L}\;(SL_{R})$: average stride length on the left (right) side.

## Data Availability

The gait data are accessible from https://reurl.cc/XAODG7 (accessed on 31 August 2025).

## Appendix A

The specifications of the trainer are available at: https://reurl.cc/oYzkXM (accessed on 31 August 2025).

## Appendix B

The comparisons of using a constant-speed and a speed-adapted treadmill are available at: https://reurl.cc/axMAZ3 (accessed on 31 August 2025).

## Appendix C

The participants’ demographic data are available at: https://reurl.cc/pYnzxb (accessed on 31 August 2025).

## Appendix D

The IMU data are accessible from https://reurl.cc/XAODG7 (accessed on 31 August 2025).

The performance index data are available at https://reurl.cc/9DdgDV (accessed on 31 August 2025).
